# Photosynthetic and Growth Response of Sugar Maple (*Acer saccharum* Marsh.) Mature Trees and Seedlings to Calcium, Magnesium, and Nitrogen Additions in the Catskill Mountains, NY, USA

**DOI:** 10.1371/journal.pone.0136148

**Published:** 2015-08-20

**Authors:** Bahram Momen, Shawna J. Behling, Greg B. Lawrence, Joseph H. Sullivan

**Affiliations:** 1 Environmental Science and Technology Department, University of Maryland, College Park, Maryland, United States of America; 2 Plant Science and Landscape Architecture Department, University of Maryland, College Park, Maryland, United States of America; 3 U.S. Geological Survey, New York Water Science Center, Troy, New York, United States of America; Technical University in Zvolen, SLOVAKIA

## Abstract

Decline of sugar maple in North American forests has been attributed to changes in soil calcium (Ca) and nitrogen (N) by acidic precipitation. Although N is an essential and usually a limiting factor in forests, atmospheric N deposition may cause N-saturation leading to loss of soil Ca. Such changes can affect carbon gain and growth of sugar maple trees and seedlings. We applied a 2^2^ factorial arrangement of N and dolomitic limestone containing Ca and Magnesium (Mg) to 12 forest plots in the Catskill Mountain region of NY, USA. To quantify the short-term effects, we measured photosynthetic-light responses of sugar maple mature trees and seedlings two or three times during two summers. We estimated maximum net photosynthesis (*An-max*) and its related light intensity (*PAR* at *An-max*), apparent quantum efficiency (*A*_*qe*_), and light compensation point (*LCP*). To quantify the long-term effects, we measured basal area of living mature trees before and 4 and 8 years after treatment applications. Soil and foliar chemistry variables were also measured. Dolomitic limestone increased Ca, Mg, and pH in the soil Oe horizon. Mg was increased in the B horizon when comparing the plots receiving N with those receiving CaMg. In mature trees, foliar Ca and Mg concentrations were higher in the CaMg and N+CaMg plots than in the reference or N plots; foliar Ca concentration was higher in the N+CaMg plots compared with the CaMg plots, foliar Mg was higher in the CaMg plots than the N+CaMg plots; *An-max* was maximized due to N+CaMg treatment; *A*_*qe*_ decreased by N addition; and *PAR* at *An-max* increased by N or CaMg treatments alone, but the increase was maximized by their combination. No treatment effect was detected on basal areas of living mature trees four or eight years after treatment applications. In seedlings, *An-max* was increased by N+CaMg addition. The reference plots had an open herbaceous layer, but the plots receiving N had a dense monoculture of common woodfern in the forest floor, which can impede seedling survival.

## Introduction

Sugar maple (*Acer saccharum* Marsh.) which is an important component of the forests of northeastern North America, has been declining for several decades [1–11]. The decline symptoms may include crown dieback, lack of regeneration, seedling mortality, poor production of samaras, reductions in growth, and tree mortality [8, 12]. Sugar maple decline may alter stand structure and long-term stability of it native habitat [13].

Sugar maple decline may partially be related to changes in soil chemistry due to acidic precipitation. Sugar maple nutrient uptake is facilitated by arbuscular mycorrhizae [14, 15], which are sensitive to soil acidification [14]. Acidic deposition can also deplete soil calcium (Ca) in eastern North America where sugar maple is common [16–20]. Sugar maple has high demand for Ca, which is required by a number of plant eco-physiological processes. Previous research indicates both negative effects of Ca depletion on [8, 10, 11, 21] and positive effects of Ca addition on sugar maple stands [9, 12, 22–24].

Ca may be supplied to the soil through weathering of parent material, decomposition of plant and animal tissues, and dry deposition of dust [25]. Most of the forest soil Ca is bound within mineral substrates that weather slowly making it unavailable for plant uptake even when it is present in large quantities. More than half of the forest's available nutrients and the fine roots that absorb them lie within the soil organic horizon and even small leaching of nutrients may hinder root nutrient uptake [26].

Decades of atmospheric deposition have also increased nitrogen (N) inputs to most of sugar maple stands. N is an essential element for plant carbon gain, and is usually a growth-limiting nutrient in terrestrial systems. However, this may no longer be the case in areas that receive high atmospheric deposition such as the Catskill Mountain region of New York [27], where continuous N deposition may cause N saturation and harm sugar maple health. This may explain the inconclusive results of linking growth and decline of sugar maple to soil N concentrations [6, 13, 28, 29]. More importantly, there has not been any study of adding N, Ca, and other basic cations such as Mg in a factorial setting to address their interactive or synergistic effects on both sugar maple seedlings and mature trees.

Changes in soil Ca, Mg, and N can affect sugar maple growth and health through a number of plant eco-physiological processes such as photosynthesis. Studies of plant photosynthetic response are usually limited to photosynthetic measurements at midday light intensities on seedlings. However, photosynthetic response at a specific light intensity does not mimic the canopy photosynthetic rate under natural condition when light intensity changes diurnally and sometimes instantly due to winds, clouds, etc. Additionally, seedling responses do not reflect mature tree responses adequately. Therefore, in this study, our main objective is to address the combined effects of a factorial combination of N and Ca+Mg treatments on important components of photosynthetic-light responses of sugar-maple seedlings and mature trees in the field. The components of photosynthetic-light response curves estimated are: maximum net photosynthesis (*An-max*), apparent quantum efficiency (*A*_*qe*_), light compensation point (*LCP*), and the light intensity at which *An-max* is attained (*PAR* at *An-max*). These characteristics convey biological and ecological importance with regard to plant growth, competitive ability, and survival, but they reveal short-term effects. To address possible treatment effects on stand growth on a longer time scale, we also measured basal area of living mature trees before and 4 and 8 years after treatment applications. Soil and foliar chemistry variables were also measured.

## Materials and Methods

### Study site

The study site was located in the Neversink River drainage (41 59' N latitude, 74 28' W longitude) in the Catskill region in southeastern New York. The environmental quality of the Catskill region is important because it serves as the primary source of drinking water for the City of New York. Catskill soils are classified mainly as Inceptisols that often have low base saturation making them susceptible to pH decreases and base cation depletion from acidic deposition [30–32]. The study plots contained a mixture of sugar maple, American beech (*Fagus grandifolia* Ehrh.) red maple (*Acer rubrum* L.) and yellow birch (*Betula alleghaniensis* Britton). The annual average temperature is 4.3 C^o^ with an annual precipitation of 175 cm, 23% of which is snowfall [31]. The study site had been selectively cut in the past, but there has been no cutting in the past 50 years. The land owner of the study site, the Frost Valley YMCA environmental education camp, granted us permission to conduct this research.

### Experimental set up

The experimental forest plots were on a hillslope along an elevational gradient. A randomized complete block design (RCBD) was used to assign treatments to experimental plots. Three blocks were set up at 670–680, 700–710, or 720–730 m elevations. Each block was subdivided into four 50- m^2^ plots for random assignment of the four treatments consisting of: 1) reference, 2) annual additions of 25 kg ha^-1^ of N from 2004–2007, 3) a single addition of 2268 kg of dolomitic limestone in fall 2003, and 4) a single addition of 2268 kg of dolomitic limestone in fall 2003 plus annual additions of 25 kg ha^-1^ of N from 2004–2007. In 2004–2005 N was added as NH_4_NO_3_, but in 2006–2007 N was added in the form of urea because NH_4_NO_3_ became commercially unavailable. The addition of 25 kg N ha^-1^ was approximately double the atmospheric deposition of N at the site. The dolomitic limestone was 17.7% Ca and 10.6% Mg, which resulted in additions of 1606 kg Ca and 962 kg Mg ha^-1^. Both the dolomite and N was applied in pelletized form by hand. To ensure uniform distribution, each plot was subdivided into 5 m^2^ subplots to receive a pre-measured dose.

### Soil sampling and analysis

Within each plot, 3 shallow pits (the primary rooting zone) were excavated to collect soil from the Oe, Oa or A, and upper B horizon before treatment applications in 2003 and 4 years later. Soil samples collected within each plot were combined by equal volume to provide one sample of each horizon per plot for chemical analysis. Samples were air-dried and sieved (1 mm), and analyzed for moisture content (oven dried at 65°C, 105°C for organic and mineral samples, respectively), exchangeable Ca^2+^, Mg^2+^, Na^+^ and K^+^ (1 M NH_4_Cl extraction), pH (0.01 M CaCl_2_ slurry and H_2_O extraction), loss-on-ignition, total N and total C (using a Thermo C/N analyzer, following USEPA methods [33]), plus exchangeable Al and H^+^ (1 M KCl extraction) following [34] except that Al was determined by inductively coupled plasma spectroscopy (ICP).

### Foliar sampling and analysis

Foliar chemistry was quantified for mature trees only to avoid harming a limited number of available seedlings. Foliage was collected by shotgun sampling of branches from the periphery of upper canopy positions from dominant or co-dominant sugar maple trees. Foliar samples were air-dried then ground and oven-dried at 70°C. Samples were digested using a microwave assisted acid digestion procedure (EPA Method 3052, 1996) then analyzed by ICP to determine Ca, Mg, and P concentrations. Total N and C were determined by Thermo C/N analyzer following USEPA methods [33].

### Photosynthetic-light curve measurements

Photosynthesis was measured on both mature trees and seedlings using a Licor Li-6400 Portable Photosynthesis System (LICOR Biosciences, Lincoln, NE) from May to July in 2005 and 2006. The red-blue LED light source was used to measure net carbon uptake at varying photosynthetically active radiation (PAR) intensities. For seedlings, photosynthetic-light response curves were measured in the field on 3–4, attached leaves of 3 randomly selected seedlings of 3 or 4 years of age in each of the 12 plots, 2 or 3 times during each summer for 2 years. Each measurement period took 4 to 5 days between 9 am and 4 pm.

Each seedling was held in complete darkness (by covering it using a piece of cloth) for 2 minutes before measurements began on to measure dark respiration first. Humidity and CO_2_ concentration were held at ambient levels, while temperature was held at the average predicted midday temperature for each sampling day. Incoming air was brought into the measurement chamber through a 2 meter-long tygon tube to prevent possible effects of the measurer's respiration on the measurement. Photosynthetic-light response curves were obtained by increasing PAR intensities until photosynthetic rate remained constant. To better represent each experimental plot, 2–3 measurements were made on each of 3–4 seedlings per plot. These multiple subsamples were averaged per plot to avoid pseudoreplication.

For mature trees, measuring photosynthetic-light responses on attached leaves was not practical particularly considering the time required to complete a set of photosynthetic-light measurements. Therefore, the measurements were made on shoots of detached branches immediately after they were obtained by shotgun sampling in the field. Photosynthetic-light measurements on detached leaves is an acceptable and common practice based on previous reports for both coniferous and hardwood species [35–39]. Moreover, possible inaccuracy of measurements on detached samples should remain similar across treatment comparisons. Shoots from mature trees were kept hydrated during measurements. Within each plot, we measured 3–4 leaves on each of 3–4 shoots that were obtained from upper canopy of each of 3–4 dominant and co-dominant trees 2 or 3 times per summer for 2 years. The data were averaged per plot to better present the photosynthetic-light responses of the dominant and co-dominant components of the stand canopy while avoiding pseudoreplication.

As an index of stand growth of the experimental plots, basal area of living, sugar maple trees with a diameter at breast height (DBH) greater than 5 cm was measured before and then 4 and 8 years after treatment application. Through time, some trees died and some new trees reached the DBH limit. Basal area data incorporate such changes.

### Statistical analysis

To model the non-linear nature of the photosynthetic-light relationship we used one of the eight parameterizations of the *Mitscherlich* function as: A_n_ = *An-max* [1-e ^(-Aqe (PAR-LCP)^] [40, 41]. In this model, A_n_ is the measured net photosynthesis at each controlled PAR intensity. *An-max* (also called *photosynthetic capacity*, *saturated An*, or *An-plateau*) is the asymptotic photosynthesis to be estimated. *A*_*qe*_ (apparent quantum efficiency) is an estimate of a scale parameter that governs the rate of change of A_n_ in response to PAR in the linear portion of the curve. *LCP* (light compensation point) is the PAR intensity at which carbon gain through photosynthesis equals carbon loss through respiration. The PAR intensity at which *An-max* is reached is another eco-physiologically important parameter that was estimated through another model called ‘*segmented linear-plateau*’ non-linear model.

Both *Mitscherlich* and *segmented linear-plateau* models were fitted using the *SAS NLIN* procedure (disregarding the repeated nature of the data) to estimate the initial parameters to be used in the *SAS NLMIXED* procedure (considering the repeated nature of data). For the *NLMIXED* procedure we also needed pooled error variance/covariance values related to the repeated nature of the data. These values were obtained using the *SAS DISCRIM* procedure. Since the *NLMIXED* procedure does not routinely produce a statistical comparison of the categorical treatment effects, we used the *ESTIMATE* statement within the *NLMIXED* procedure to compare main, interactive, and simple effects of N and Ca+Mg treatments (*SAS* codes and related data are provided in S1 Table). The *SAS MIXED* procedure was used to perform analysis of covariance on the basal area data (provided in S2 Table). The standing basal areas of living sugar maple trees before treatment applications were not homogenous, and it was thus, used as a covariate for the analysis of basal area data 4 or 8 years after treatment applications. The *SAS MIXED* procedure was used to perform analysis of variance on the foliar and soil chemistry data.

## Results

### Soil and Foliar Chemistry

Prior to treatments, no significant difference existed among experimental plots for soil chemistry variables studied. Compared with the reference plots, plots that received a total of 100 kg N in 4 years did not differ significantly for any of the soil responses measured (Table 1).

**Table 1:** Means (standard errors) of soil chemistry variables in Oe, Oa and B horizons in reference plots, and plots that received nitrogen (N), dolomitic limestone (CaMg) and nitrogen plus dolomitic limestone (N+CaMg). Different superscripts (a, b) show mean differences within a horizon (P < 0.05).

Soil Response	Horizon	Reference	N	CaMg	N+CaMg
Ca (cmol_c_ kg^-1^)	Oe	11.9^**a**^ (1.78)	10.7^**a**^ (3.29)	41.4^**b**^ (8.14)	39.4^**b**^ (7.27)
	Oa/A	1.80 (0.48)	2.60 (1.67)	18.00 (9.06)	9.20 (4.91)
	B	0.44 (0.08)	0.17 (0.06)	1.01 (0.21)	0.64 (0.31)
Mg (cmol_c_ kg^-1^)	Oe	2.8^**a**^ (0.38)	2.6^**a**^ (0.55)	22.4^**b**^ (5.89)	20.8^**b**^ (3.18)
	Oa/A	0.67 (0.12)	0.74 (0.34)	13.7 (6.11)	7.6 (4.5)
	B	0.18^**ab**^ (0.04)	0.10^**a**^ (0.01)	1.08^**b**^ (0.24)	0.60^**ab**^ (0.12)
Al (cmol_c_ kg^-1^)	Oe	3.8 (0.31)	4.6 (1.96)	1.9 (0.51)	2.3 (0.39)
	Oa/A	9.3 (5.02)	7.6 (3.40)	1.9 (1.60)	3.6 (0.92)
	B	5.3 (0.92)	5.2 (1.79)	3.4 (0.21)	5.1 (0.81)
Organic C (g kg^-1^)	Oe	427 (28)	505 (12)	452 (19)	341 (43)
	Oa/A	174 (8)	226 (7)	210 (7)	203 (7)
	B	41.7 (14)	29.0 (12)	20.3 (2)	30.4 (9)
Total N (g kg^-1^)	Oe	23.1 (1.5)	25.5 (1)	24.2 (1.5)	18.6 (2.6)
	Oa/A	9.1 (3.5)	11.7 (2.9)	10.5 (4.0)	11.4 (4.0)
	B	2.6 (0.8)	2.0 (0.7)	1.5 (0.2)	2.1 (0.5)
Organic Matter (g kg^-1^)	Oe	775 (40)	881 (32)	686 (21)	697 (84)
	Oa/A	251 (77)	388 (101)	362 (116)	329 (108)
	B	92.7 (27)	59.1 (23)	69.5 (26)	52.9 (7)
pH	Oe	3.65^**a**^ (0.06)	3.50^**a**^ (0.11)	5.16^**b**^ (0.42)	4.97^**b**^ (0.40)
	Oa/A	3.84 (0.12)	3.72 (0.14)	4.74 (0.35)	4.46 (0.23)
	B	3.97 (0.19)	4.03 (0.08)	4.30 (.28)	4.33 (0.09)

The addition of limestone significantly (P < 0.05) increased Ca, Mg, and pH in the Oe horizon (Table 1). Mg was increased in the B horizon by CaMg only when comparing the plots receiving N with those receiving CaMg.

No significant difference (i.e., P > 0.05) was detected for foliar N, C, or P concentration of sugar maple trees between the reference and treatment plots. (Table 2).

**Table 2:** Means (standard errors) of foliar chemistry of sugar maple trees in reference plots, and plots that received nitrogen (N), dolomitic limestone (CaMg) and dolomitic limestone plus nitrogen (N+CaMg). Treatment plots were replicated three times, but 8 to 13 trees were subsampled. Different superscripts (a, b, c) show mean differences within a horizon (P < 0.05).

Foliar response	Reference	N	CaMg	N+CaMg
N (g kg^-1^)	22.4 (1.10)	23.6 (0.81)	22.7 (1.04)	24.3 (1.15)
C (g kg^-1^)	475 (3.29)	479 (4.21)	469 (2.25)	465 (1.91)
P (g kg^-1^)	1.22 (0.08)	1.25 (0.08)	1.35 (0.08)	1.35 (0.08)
Ca (g kg^-1^)	4.78^**a**^ (1.15)	3.38^**a**^ (0.41)	6.63^**b**^ (1.04)	9.17^**c**^ (1.27)
Mg (g kg^-1^)	1.54^**a**^ (0.29)	1.27^**a**^ (1.15)	2.66^**c**^ (0.38)	2.14^**b**^ (0.28)

Foliar Ca and Mg concentrations were significantly higher (P < 0.05) in the CaMg and N+CaMg plots than in the reference or N plots (Table 2) indicating that dolomitic limestone application increased Ca and Mg uptake and allocation to leaves. Foliar Ca concentration was higher in the N+CaMg plots compared with the CaMg plots, but foliar Mg was higher in the CaMg plots than the N+CaMg plots.

### Photosynthetic-light responses and basal area

Preliminary analysis of photosynthetic data indicated no significant monthly or yearly effect for mature trees or seedlings. Therefore, the data collected from May to July of both 2005 and 2006, as well as several subsamples within each measuring time, were averaged per plot (replicate) prior to statistical analyses. Photosynthetic data are analyzed and reported for mature trees and seedlings separately.

### Seedlings

**Fig 1:**
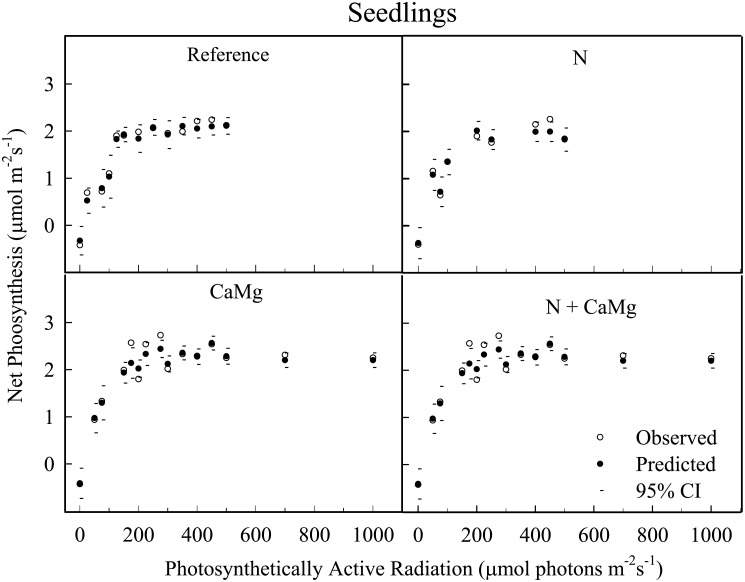
Photosynthetic-light responses of sugar maple seedlings. Predicted and confidence interval (CI) values are based on the *Mitscherlich* function. The observed values are means of 3 replicates per treatment with subsample measurements on multiple leaves on multiple seedlings 2 or 3 times per summer during 2 years within each plot.

The *Mitscherlich* function fitted seedling data very well as indicated by the closeness of the measured (observed) and predicted values and their narrow confidence intervals (Fig 1).

The lowest *An-max* value was observed due to N addition alone, but the decrease was not statistically significant (i.e., P > 0.05 (Table 3). However, N addition magnified the effect of CaMg addition to increase *An-max* significantly (Table 3). *A*_*qe*,_ and *LCP* did not differ significantly among the treatments. *PAR* at *An-max* was highest in the N+CaMg plots, but the increase was not statistically significant.

**Table 3:** Photosynthetic-light parameter estimates (*An-max*, *A*_*qe*,_ *LCP*, and *PAR at An-plateau*) and their standard errors for sugar maple seedlings in the reference and treatment plots. Estimated parameters are based on 3 replicates per treatment with subsample measurements of multiple leaves on multiple seedlings 2 or 3 times per summer during 2 years. Different superscripts (a, b) show treatment mean differences within a row (P < 0.05).

Estimate		Treatment		
	Reference	N	CaMg	N+CaMg
*An-max* (μmol m^-2^ s^-1^)	2.13^**a**^ (0.26)	1.81^**a**^ (0.22)	2.24^**ab**^ (0.22)	2.88^**b**^ (0.21)
*A*_*qe*_	0.015 (0.001)	0.016 (0.001)	0.017 (0.001)	0.015 (0.002)
*LCP* (μmol m^-2^ s^-1^)	111 (51)	98 (40)	97 (42)	160 (40)
*PAR at An-max* (μmol m^-2^ s^-1^)	168 (38)	178 (16)	157 (18)	204 (14)

### Mature trees

**Fig 2:**
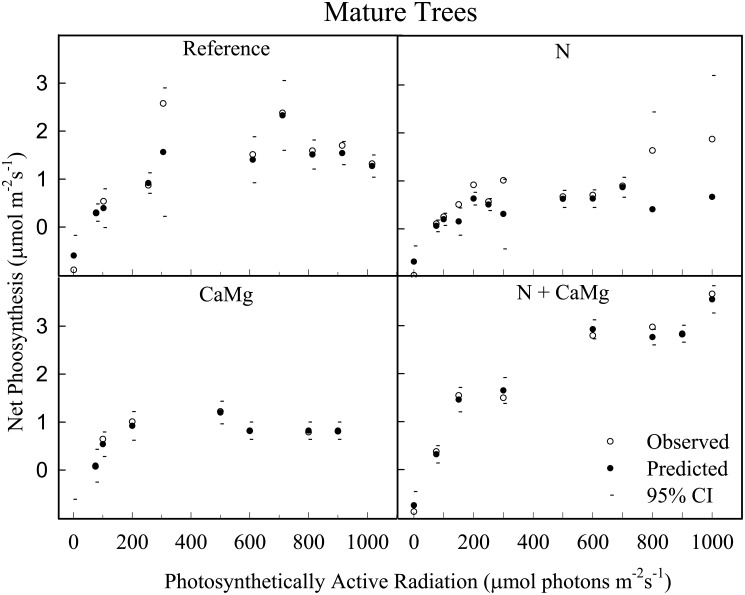
Photosynthetic-light responses of sugar maple trees. Predicted and confidence interval (CI) values are based on the Mitscherlich function. The observed values are means of 3 replicates per treatment with subsamples measurements on multiple leaves on multiple shoots from different parts of canopy of dominant and co-dominant trees 2 or 3 times per summer during 2 years within each plot.

The *Mitscherlich* function fitted mature tree data adequately (Fig 2).

*An-max* was increased (P < 0.01) by the N+CaMg treatment compared to each of the other three treatments (Table 4). *PAR* at *An-max* was increased by N or CaMg addition alone, but the increase was most pronounced under their combination (i.e., N+CaMg treatment). *A*_*qe*_ decreased (P < 0.01) by N both in the presence or absence of CaMg application. *LCP* was not affected significantly by the treatments.

**Table 4:** Photosynthetic-light parameter estimates (*An-max*, *A*_*qe*,_ *and LCP*) and their standard errors of sugar maple mature trees in the reference and treatment plots. Estimated parameters are based on 3 replicates per treatment with subsample measurements of multiple leaves on multiple canopy locations 2 or 3 times per summer during 2 years. Different superscripts (a, b, c) show treatment mean differences within a row (P < 0.05).

Estimate		Treatment		
	Reference	N	CaMg	N+CaMg
*An-max* (μmol m^-2^ s^-1^)	1.2^**a**^ (.26)	1.5^**a**^ (0.3)	0.7^**a**^ (0.5)	3.4^**b**^ (0.4)
*A*_*qe*_	0.011^**b**^ (.001)	0.006^**a**^ (0.001)	0.015^**b**^ (0.002)	0.004^**a**^ (0.001)
*LCP* (μmol m^-2^ s^-1^)	50.90 (9.7)	72.38 (11.4)	63.05 (12.3)	50.36 (14.2)
*PAR at An-max* (μmol m^-2^ s^-1^)	210^**a**^ (71)	422^**b**^ (22)	409^**b**^ (46)	630^**c**^ (34)

**Fig 3:**
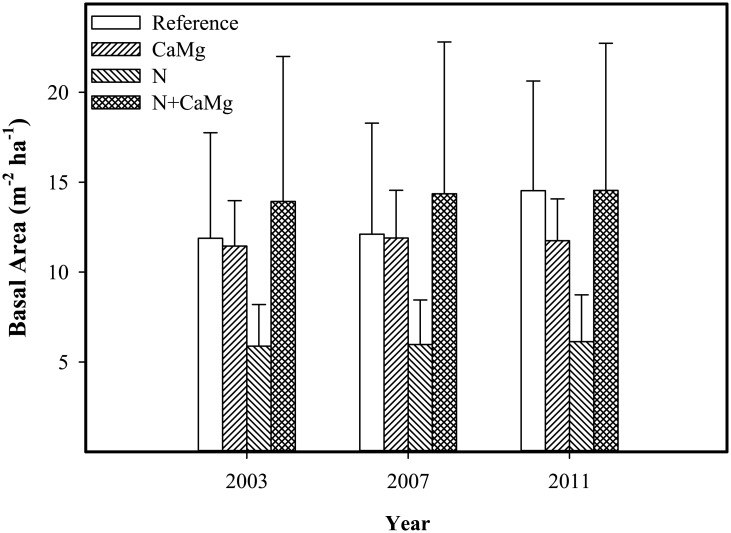
Mean basal area (standard error) of living mature sugar maple trees with DBH > 5 cm before treatment applications in 2003 and thereafter. Treatment means do not differ within or across time.

Basal area of living sugar maple trees in the study plots were similar before treatment applications and were not affected (i.e., P > 0.05) by treatments 4 or 8 years after treatment applications (Fig 3).

## Discussion

### Soil Chemistry

Detectable differences in soil measurements between the reference and treatment plots were limited mostly to the Oe horizon. However, the added nutrients were most likely available for plant use because Oe is the horizon where fine root density is greatest. Soil total N measurements did not reveal any effect from N additions perhaps because the annual additions of 25 kg N ha^-1^ were too small (1–3%) relative to the existing soil N pool reported for a nearby watershed in the Neversink River basin [42]. Moreover, soil pH measurements did not indicate that the N additions increased nitrification sufficiently to decrease soil pH (Table 1). Results from adding CaMg alone or in combination with N were similar in the Oe horizon (Table 1), suggesting the lack of significant interactive or synergistic effects of N and CaMg on soil variables measured. This is consistent with previous reports [9, 22].

### Foliar chemistry of mature trees

**Fig 4:**
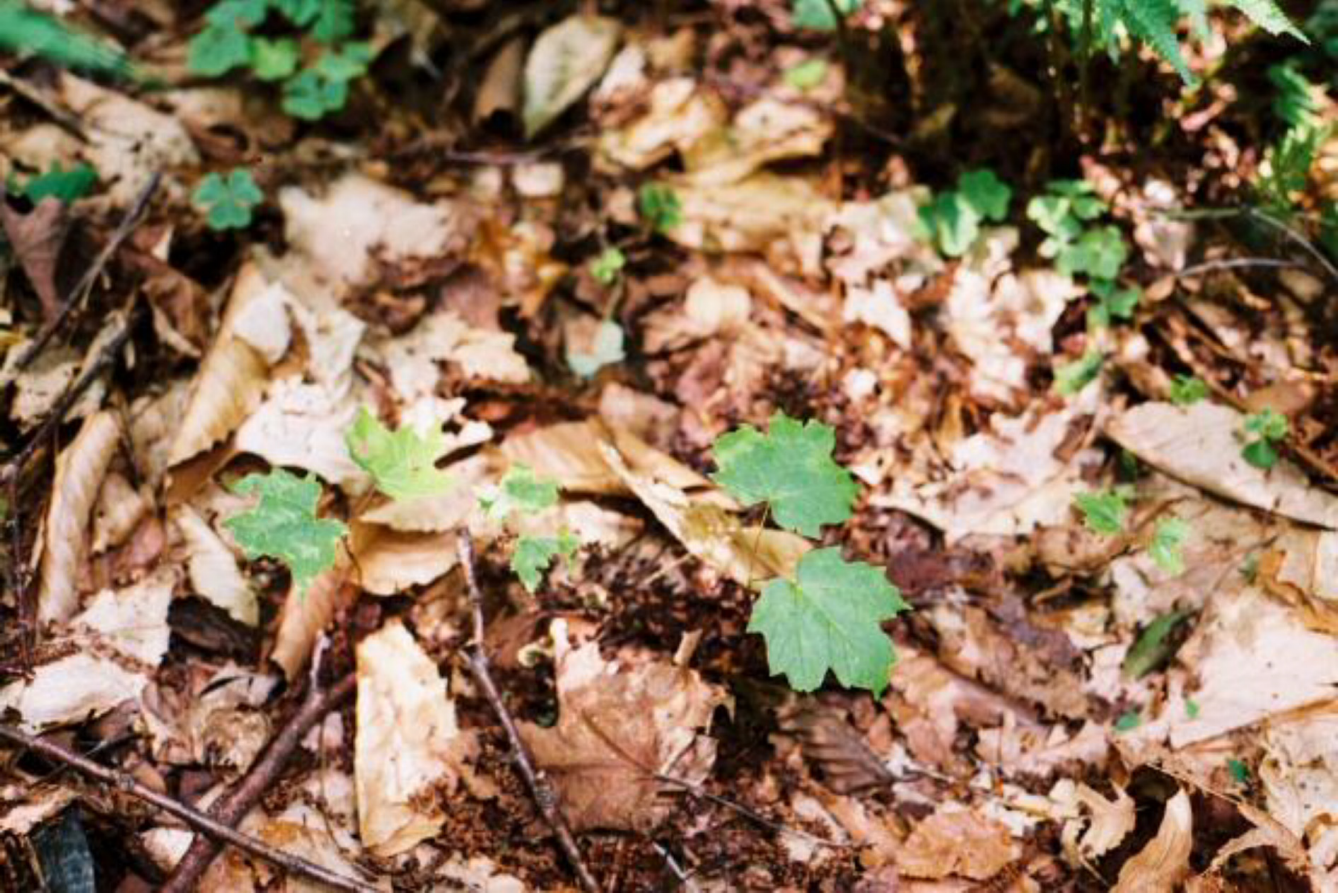
Sugar maple seedlings in a reference plot.

**Fig 5:**
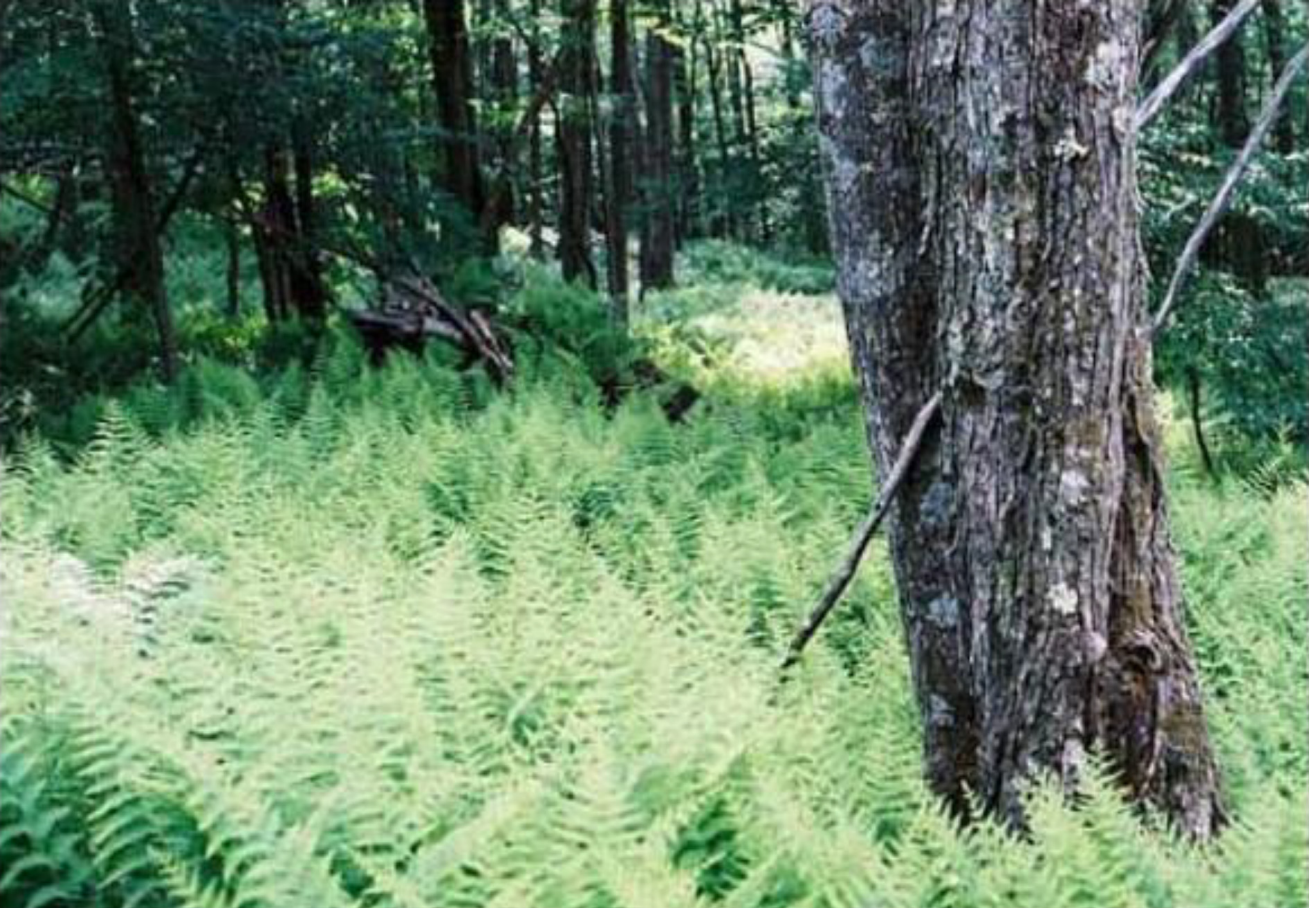
Dense presence of common woodfern (*Dryopteris intermedia* Muhl. ex Willd.) in a plot receiving N.

Foliar N did not respond to N addition. This differs from previous reports of increased foliar N concentrations in sugar maple trees due to increased N inputs [43, 44]. Plant N dynamics depends on whether or not soil N is a limiting factor. A previous report [45] indicates that our study site might have had a large excess of plant-available N (i.e., N saturated) prior to N fertilization, and thus, further increases in soil N availability due to experimental N application could not affect foliar N content. The added N could have been taken from the soil (hence no increase in soil N) to increase the growth of mature trees without increasing their foliar N content. However, the growth of mature trees, as indicated by their basal area, was not affected by the added N. Added N could be assimilated by understory species such as ferns, but previous research on this topic is both limited and inconclusive. A major understory species in our study site is common woodfern (*Dryopteris intermedia* Muhl. ex Willd.), and N addition did not affect its relative growth rate in our experimental plots [46], or its ground cover in a comparable forest site in the Adirondack Park, NY [47]. However, in California's San Bernardino Mountains N addition increased biomass of bracken fern in high pollution but not in low pollution sites [48]. Although, we did not focus on statistical evaluation of treatment effects on understory species, we subjectively observed that the reference plots had a relatively open herbaceous layer (Fig 4), whereas the plots that received N had a substantially higher density of common woodfern (Fig 5). However, this difference seemed to exist before treatment applications and the possible differential rates of change due to treatment applications merits further research.

Foliar Ca concentration was higher in the CaMg+N plots compared with the CaMg plots, but foliar Mg was higher in the CaMg plots than the CaMg+N plots. If real, such reverse effects indicates differing role of N in Ca and Mg uptake and allocation, which merits further investigation.

### Photosynthetic-light responses

*An-max* of seedlings was increased by CaMg addition and this increase was augmented by N addition. For seedlings, *PAR* at *An-max* was also highest in the N+CaMg plots although not significantly. These results suggest that 1) a slight increase in *PAR* can facilitate a significant increase in *An-max of* seedlings under N+CaMg treatment, and 2) possible negative effects of continuous atmospheric N addition on potential photosynthesis may be overcompensated by CaMg addition. However, such potential photosynthesis requires increased light intensities under the canopy, which may not be feasible if the density of ferns is increased by continuous atmospheric N input. In fact, we observed many seedlings that were thriving in early summer but died of overcrowding by August. Although sugar maple seedlings are considered shade tolerant, they still require some light for carbon gain, and a dense fern cover might prevent sufficient penetration of light to sustain the seedlings.

For mature trees, both *An-max* and its related *PAR* intensity were highest under the N+CaMg treatment suggesting that under continuous atmospheric N addition, CaMg addition might enable mature trees to use more of the available light in the upper canopy to attain higher photosynthetic saturation rates.

*A*_*qe*_ of mature trees was decreased by N addition and this decrease was not compensated by CaMg addition. *A*_*qe*_ is related to the efficiency of carbon gain at low light intensities, and the negative effect of N addition on such efficiency may not be detrimental for mature trees that form the upper stand canopy and are not as light-limited as seedlings.

Although N is a major requirement for photosynthesis, N addition alone did not enhance photosynthetic-light responses of seedlings or mature trees. This is consistent with a previous report of no correlation between nitrogen additions and photosynthesis [43]. This lack of N effect can be expected considering the aforementioned lack of N effect on soil or foliar N chemistry.

The effects of dolomitic limestone on sugar maple growth, health, and vigor have been studied widely [12, 18, 49]. However, we have found no previous reports of the photosynthetic-light responses of both seedlings and mature trees to dolomitic limestone and N in the field for a more through comparison. In general, dolomitic limestone addition had a number of positive effects on photosynthetic-light relationships of sugar maple, but the effects were not similar for seedlings and mature trees. The few positive short-term effects on the photosynthetic responses of mature-trees were not sufficient to increase the basal area of sugar maple mature trees. This is not surprising because increased instantaneous photosynthesis does not necessarily lead to an increase in net primary productivity (NPP) and even with increased NPP, additional fixed carbon may not be allocated to basal area growth. We should point out that the long-term growth and health of sugar maple stands depends both on seedling regeneration and survival and on growth dynamics of sugar maple trees in relation to other under- and over-story species within the forest stands. Therefore, additional ecological and physiological research is needed to better understand sugar maple decline.

## Supporting Information

S1 Table Photosynthetic-light data for sugar maple seedlings and mature trees, and SAS codes to perform non-linear analysis of treatment effects. (PDF)

S2 Table Basal area data for living mature sugar maple trees in 2003, 2007, and 2011. (PDF)
