# Supplementary material for: Photosynthetic and Growth Response of Sugar Maple (Acer saccharum Marsh.) Mature Trees and Seedlings to Calcium, Magnesium, and Nitrogen Additions in the Catskill Mountains, NY, USA
Source: PLoS One. 2015 Aug 20;10(8):e0136148. doi: 10.1371/journal.pone.0136148 (PMC4546351; doi:10.1371/journal.pone.0136148)
Supplement: S1 Table — (PDF) [file pone.0136148.s001.pdf]

S1 Table. Photosynthetic-light data, averaged per plot, for seedlings and mature sugar maple trees with related SAS codes to perform non-linear analysis of treatment efects.

```
data a;
input age$ plot trt$ par an;
if Trt='A'  then do; za=1; zb=0; zc=0; zd=0; end; *includes A;
 if Trt='B' then do; za=0; zb=1; zc=0; zd=0; end; *includes B;
 if Trt='C' then do; za=0; zb=0; zc=1; zd=0; end; *includes C;
 if Trt='D' then do; za=0; zb=0; zc=0; zd=1; end; *includes D;
 /*Trt=A when Ca='n' and N='n'
   Trt=B when Ca='n' and N='y'
   Trt=C when Ca='y' and N='n'
   Trt=D when Ca='y' and N='y'*/
cards;
Seedling 1 D 0 -5.60
Seedling 1 D 50 14.67
Seedling 1 D 100 19.70
Seedling 1 D 200 24.10
Seedling 1 D 300 28.20
Seedling 1 D 350 25.20
Seedling 1 D 400 29.50
Seedling 1 D 500 25.90
Seedling 2 B 0 -3.39
Seedling 2 B 50 10.90
Seedling 2 B 200 16.70
Seedling 2 B 250 17.80
Seedling 2 B 400 21.50
Seedling 2 B 450 22.60
Seedling 3 C 0 -2.96
Seedling 3 C 50 12.80
Seedling 3 C 200 18.00
Seedling 3 C 300 21.25
Seedling 3 C 400 21.77
Seedling 3 C 500 20.63
Seedling 4 A 0 -4.45
Seedling 4 A 25 6.92
Seedling 4 A 125 19.00
Seedling 4 A 150 19.06
Seedling 4 A 200 18.55
Seedling 4 A 250 20.65
Seedling 4 A 350 19.90
Seedling 4 A 450 22.00
Seedling 4 A 500 22.84
Seedling 5 D 0 -2.34
Seedling 5 D 75 12.26
Seedling 5 D 150 18.32
Seedling 5 D 200 21.43
Seedling 5 D 250 23.53
Seedling 5 D 500 20.73
Seedling 5 D 700 28.95
Seedling 6 B 0 -5.34
Seedling 6 B 50 12.28
Seedling 6 B 100 18.30
Seedling 6 B 200 21.25
Seedling 6 B 250 21.05
Seedling 6 B 500 22.20
Seedling 7 A 0 -3.93
Seedling 7 A 75 7.19
Seedling 7 A 100 11.00
Seedling 7 A 200 21.10
```

Seedling 7 A 300 19.53
Seedling 7 A 400 22.10
Seedling 7 A 450 22.80
Seedling 7 A 500 19.60
Seedling 8 C 0 -4.60
Seedling 8 C 75 13.47
Seedling 8 C 150 19.89
Seedling 8 C 300 19.10
Seedling 8 C 350 21.60
Seedling 8 C 400 22.90
Seedling 8 C 500 21.78
Seedling 8 C 700 23.10
Seedling 8 C 1000 22.50
Seedling 9 D 0 -14.51
Seedling 9 D 100 23.60
Seedling 9 D 500 35.15
Seedling 11 B 0 -3.30
Seedling 11 B 75 6.46
Seedling 11 B 100 8.82
Seedling 11 B 250 14.10
Seedling 11 B 500 14.65
Seedling 12 C 0 -5.31
Seedling 12 C 50 5.93
Seedling 12 C 75 13.13
Seedling 12 C 175 25.70
Seedling 12 C 225 25.40
Seedling 12 C 275 27.30
Seedling 12 C 350 25.00
Seedling 12 C 400 24.03
Seedling 12 C 450 25.40
Seedling 12 C 500 25.02
Tree 2 B 0 -3.37
Tree 2 B 75 2.11
Tree 2 B 150 5.66
Tree 2 B 300 14.20
Tree 2 B 800 25.30
Tree 2 B 1000 25.70
Tree 4 A 0 -9.30
Tree 4 A 100 1.91
Tree 4 A 600 15.10
Tree 4 A 800 15.90
Tree 4 A 900 17.00
Tree 4 A 1000 16.30
Tree 5 D 0 -10.13
Tree 5 D 75 2.33
Tree 5 D 300 14.95
Tree 5 D 600 23.90
Tree 5 D 800 29.70
Tree 5 D 900 28.20
Tree 6 B 0 -11.35
Tree 6 B 75 -0.01
Tree 6 B 100 3.53
Tree 6 B 200 12.20
Tree 6 B 700 10.02
Tree 6 B 1000 11.69
Tree 7 A 0 -9.32
Tree 7 A 75 2.91

```
Tree 7 A 100 5.93
Tree 7 A 250 8.69
Tree 7 A 1000 10.09
Tree 8 C 0 -15.51
Tree 8 C 75 0.75
Tree 8 C 200 10.06
Tree 8 C 500 12.20
Tree 9 D 0 -7.40
Tree 9 D 75 5.14
Tree 9 D 150 15.43
Tree 9 D 600 31.93
Tree 9 D 1000 36.60
Tree 10 A 0 -8.21
Tree 10 A 100 8.22
Tree 10 A 300 25.75
Tree 10 A 700 23.77
Tree 11 B 0 -14.38
Tree 11 B 100 1.49
Tree 11 B 150 4.47
Tree 11 B 200 6.12
Tree 11 B 250 5.66
Tree 11 B 300 6.01
Tree 11 B 500 6.70
Tree 11 B 600 7.02
Tree 11 B 700 7.89
Tree 11 B 800 7.36
Tree 12 C 0 -12.10
Tree 12 C 100 6.43
Tree 12 C 600 8.16
Tree 12 C 800 7.88
Tree 12 C 900 8.08
;

/*
option ps=20 ls=64;
proc plot data=a;
plot an*par='.';*haxis= 0 to 1000 by 100 vaxis= -5 to 35 by 10;
by age plot;
quit;
*/
/*
*Mitscherlich model;
Proc nlin data=a; * to generate estimates of parameters per plot;
parms Amax=25 Slp=10 Lcp=30;
      Variance=.555;
      e= exp(-.001*slp*(Par-Lcp)); *-.001 s used to ease convergance.
Slp values  indicated as Parameter and then estimated are 1000 greater;
      F=Amax*(1-e);
model An =F;
by age plot;
quit;
*/
/*
data individual; *including parameter estimates from above;
input age$ plot trt$ Anmax Slp Lcp MSe;
lines;
Seed  1 D 26.82 17.06 10.46 3.98
```

```
Seed  2 B 20.28 15.38 8.72 6.48
Seed  3 C 20.57 21.22 6.16 2.36
Seed  4 A 20.80 21.35 8.41 1.86
Seed  5 D 25.26  9.68 8.82 8.33
Seed  6 B 21.69 20.92 10.49 .17
Seed  7 A 22.14 9.25 20.10 4.18
Seed  8 C 22 15.25 12.41 1.62
Seed  9 D 35.18 14.56 23.71 .
Seed 11 B 15 10.69 18.95 .16
Seed 12 C 26.18 12.56 17.61 4.47
Tree  2 B 28.89 2.54 47.01 .73
Tree  4 A 16.46 5.59 79.67 .27
Tree  5 D 30.74 3.28 75.99 4.78
Tree  6 B 11.64 11.22 62.55 4.9
Tree  7 A 9.74 14.58 46.11 .51
Tree  8 C 12.42 11.83 68.62 .12
Tree  9 D 35.94 4.7 39.46 2.03
Tree 10 A 25.8 7.88 37.1 17.59
Tree 11 B 7.07 13.54 81.93 .3
Tree 12 C 8.04 25.26 36.35 .02
RUN;
PROC SORT DATA=InDIVIDUAL;
BY AGE TRT;
RUN;
proc print data=individual;
run;
PROC MEANS DATA = INDIVIDUAL MEAN VAR;
VAR Anmax Slp Lcp MSe;
BY AGE trt;
RUN;
proc discrim data=individual pcov bcov;
class trt;
var Anmax Slp Lcp;
by age;
run;*/
data seed;
set a;
if age='Seedling';
run;
data tree;
set a;
if age='Tree';
run;

proc sort data=seed;
by plot;
run;
Proc nlmixed data=seed method=firo;
parms AmaxA=22  AmaxB=18 AmaxC=23 AmaxD=30
   SlpA=15   SlpB=16  SlpC=16 SlpD=13 LcpA=14  LcpB=13  LcpC=12 LcpD=15
var=3;
      Amax=(AmaxA*za)+(AmaxB*zb)+(AmaxC*zc)+(AmaxD*zd)+u1;
      Slp= (SlpA*za)+(SlpB*zb)+(SlpC*zc)+(SlpD*zd)+u2;
      Lcp= (LcpA*za)+(LcpB*zb)+(LcpC*zc)+(LcpD*zd)+u3;
      e= exp(-.001*slp*(Par-Lcp));
      F=Amax*(1-e);
model An ~ normal(F,var);* var of An for seedlings across trt;
```

```
   random u1 u2 u3 ~ normal([0,0,0],[14,2.25,27.59,13,-
20.75,46.74])subject=plot;
   predict F out=predseed;
   /*run;
   proc print data=predseed;
   var plot trt par an pred lower upper;
   run;
   proc sort data=predseed;
   by trt par;
   run;
   proc means data=predseed mean noprint;
   var an pred lower upper;
   by trt par;
   output out=seedGr mean=;
   run;*/

   /*proc print data=seedGr;
   var trt par an pred lower upper;
   run;
   */
      *Anmax interactive, main, and simple effect contrasts;
   estimate 'Interaction on Amax' (Amaxb-Amaxa)/2 - (Amaxd-Amaxc)/2;

    estimate 'Main Mean of Amax w Ca'   (Amaxc+Amaxd)/2;
    estimate 'Main Mean of Amax wo Ca'  (Amaxa+Amaxb)/2;
    estimate 'Ca Effect on Amax'   (Amaxc+Amaxd)/2 - (Amaxa+Amaxb)/2;

    estimate 'Main Mean of Amax w N'  (Amaxb+Amaxd)/2;
    estimate 'Main Mean of Amax wo N' (Amaxa+Amaxc)/2;
    estimate 'N Effect on Amax'    (Amaxb+Amaxd)/2 - (Amaxa+Amaxc)/2;

    estimate 'Amaxa-Amaxb' Amaxa-Amaxb;
    estimate 'Amaxa-Amaxc' Amaxa-Amaxc;
    estimate 'Amaxa-Amaxd' Amaxa-Amaxd;
    estimate 'Amaxb-Amaxc' Amaxb-Amaxc;
    estimate 'Amaxb-Amaxd' Amaxb-Amaxd;
    estimate 'Amaxc-Amaxd' Amaxc-Amaxd;

         *Slope interactive, main, and simple effect contrasts;
    estimate 'Interaction on Slp' (slpb-slpa)/2 - (slpd-slpc)/2;
    estimate 'Main Mean of slp w Ca'  (slpc+slpd)/2;
    estimate 'Main Mean of slp wo Ca' (slpa+slpb)/2;
    estimate 'Ca Effect on Slp'   (slpc+slpd)/2 - (slpa+slpb)/2;

    estimate 'Main Mean of slp w N'  (slpb+slpd)/2;
    estimate 'Main Mean of slp wo N' (slpa+slpc)/2;
    estimate 'N Effect on Slp'    (slpb+slpd)/2 - (slpa+slpc)/2;

    estimate 'slpa-slpb' slpa-slpb;
    estimate 'slpa-slpc' slpa-slpc;
    estimate 'slpa-slpd' slpa-slpd;
    estimate 'slpb-slpc' slpb-slpc;
    estimate 'slpb-slpd' slpb-slpd;
    estimate 'slpc-slpd' slpc-slpd;

        *LCP interactive, main, and simple effect contrasts;
    estimate 'Interaction on LCP' (Lcpb-Lcpa)/2 - (Lcpd-Lcpc)/2;
```

```
     estimate 'Main Mean of LCP w Ca'  (Lcpc+Lcpd)/2;
     estimate 'Main Mean of LCP wo Ca' (Lcpa+Lcpb)/2;
     estimate 'Ca Effect on LCP'   (Lcpc+Lcpd)/2 - (Lcpa+Lcpb)/2;

     estimate 'Main Mean of slp w N'  (Lcpb+Lcpd)/2;
     estimate 'Main Mean of slp wo N' (Lcpa+Lcpc)/2;
     estimate 'N Effect on LCP'    (Lcpb+Lcpd)/2 - (Lcpa+Lcpc)/2;
     estimate 'Lcpa-Lcpb' Lcpa-Lcpb;
     estimate 'Lcpa-Lcpc' Lcpa-Lcpc;
     estimate 'Lcpa-Lcpd' Lcpa-Lcpd;
     estimate 'Lcpb-Lcpc' Lcpb-Lcpc;
     estimate 'Lcpb-Lcpd' Lcpb-Lcpd;
     estimate 'Lcpc-Lcpd' Lcpc-Lcpd;
    quit;
    proc sort data=tree;
    by plot;
    run;
    Proc nlmixed data=tree method=firo;
    parms AmaxA=17  AmaxB=15 AmaxC=11 AmaxD=33
          SlpA=9 SlpB=9  SlpC=15 SlpD=4 LcpA=54  LcpB=64  LcpC=57 LcpD=55
var=3;
          Amax=(AmaxA*za)+(AmaxB*zb)+(AmaxC*zc)+(AmaxD*zd)+u1;
          Slp= (SlpA*za)+(SlpB*zb)+(SlpC*zc)+(SlpD*zd)+u2;
          Lcp= (LcpA*za)+(LcpB*zb)+(LcpC*zc)+(LcpD*zd)+u3;
          e= exp((-0.001*slp)*(Par-Lcp));
          F=Amax*(1-e);
    model An ~ normal(f,var);
    random u1 u2 u3 ~ normal([0,0,0],[69.7,-34.57,33.68,-83.79,-
27.93,467.77])subject=plot;
    predict F out=predtree;
    /*run;
    proc print data=predtree;
    var plot trt par an pred lower upper;
    run;
    proc sort data=predtree;
    by trt par;
    run;
    proc means data=predtree mean noprint;
    var an pred lower upper;
    by trt par;
    output out=TreeGr mean=;
    run;
    proc sort data=TreeGr;
    by trt par;
    run;
    proc print data=TreeGr;
    var trt par an pred lower upper;
    run;
    */
    *Anmax interactive, main, and simple effect contrasts;
    estimate 'Interaction on Amax' (Amaxb-Amaxa)/2 - (Amaxd-Amaxc)/2;

     estimate 'Main Mean of Amax w Ca'   (Amaxc+Amaxd)/2;
     estimate 'Main Mean of Amax wo Ca'  (Amaxa+Amaxb)/2;
     estimate 'Ca Effect on Amax'   (Amaxc+Amaxd)/2 - (Amaxa+Amaxb)/2;
```

```
 estimate 'Main Mean of Amax w N'  (Amaxb+Amaxd)/2;
 estimate 'Main Mean of Amax wo N' (Amaxa+Amaxc)/2;
 estimate 'N Effect on Amax'    (Amaxb+Amaxd)/2 - (Amaxa+Amaxc)/2;

 estimate 'Amaxa-Amaxb' Amaxa-Amaxb;
 estimate 'Amaxa-Amaxc' Amaxa-Amaxc;
 estimate 'Amaxa-Amaxd' Amaxa-Amaxd;
 estimate 'Amaxb-Amaxc' Amaxb-Amaxc;
 estimate 'Amaxb-Amaxd' Amaxb-Amaxd;
 estimate 'Amaxc-Amaxd' Amaxc-Amaxd;

 *Slope interactive, main, and simple effect contrasts;
 estimate 'Interaction on Slp' (slpb-slpa)/2 - (slpd-slpc)/2;
 estimate 'Main Mean of slp w Ca'  (slpc+slpd)/2;
 estimate 'Main Mean of slp wo Ca' (slpa+slpb)/2;
 estimate 'Ca Effect on Slp'   (slpc+slpd)/2 - (slpa+slpb)/2;

 estimate 'Main Mean of slp w N'  (slpb+slpd)/2;
 estimate 'Main Mean of slp wo N' (slpa+slpc)/2;
 estimate 'N Effect on Slp'    (slpb+slpd)/2 - (slpa+slpc)/2;

 estimate 'slpa-slpb' slpa-slpb;
 estimate 'slpa-slpc' slpa-slpc;
 estimate 'slpa-slpd' slpa-slpd;
 estimate 'slpb-slpc' slpb-slpc;
 estimate 'slpb-slpd' slpb-slpd;
 estimate 'slpc-slpd' slpc-slpd;

 *LCP interactive, main, and simple effect contrasts;
 estimate 'Interaction on LCP' (Lcpb-Lcpa)/2 - (Lcpd-Lcpc)/2;

 estimate 'Main Mean of LCP w Ca'  (Lcpc+Lcpd)/2;
 estimate 'Main Mean of LCP wo Ca' (Lcpa+Lcpb)/2;
 estimate 'Ca Effect on LCP'   (Lcpc+Lcpd)/2 - (Lcpa+Lcpb)/2;

 estimate 'Main Mean of slp w N'  (Lcpb+Lcpd)/2;
 estimate 'Main Mean of slp wo N' (Lcpa+Lcpc)/2;
 estimate 'N Effect on LCP'    (Lcpb+Lcpd)/2 - (Lcpa+Lcpc)/2;
 estimate 'Lcpa-Lcpb' Lcpa-Lcpb;
 estimate 'Lcpa-Lcpc' Lcpa-Lcpc;
 estimate 'Lcpa-Lcpd' Lcpa-Lcpd;
 estimate 'Lcpb-Lcpc' Lcpb-Lcpc;
 estimate 'Lcpb-Lcpd' Lcpb-Lcpd;
 estimate 'Lcpc-Lcpd' Lcpc-Lcpd;
quit;

/*linear-plataeu model to estimate Par at Anmax;
proc sort data=a;
by age plot;
quit;

Proc nlin data=a; * to generate estimates of parameters per plot;
 parms a=-5 b=.2 x0=200;
  if (Par<x0) then mean=a+b*Par*(1-Par/(2*x0));
              else mean=a+b*x0/2;
  model An=mean;
 by age plot;
```

```
*output out=b predicted=yp;
quit;

data ind1; *including parameter estimates from above;
input age$ plot trt$ a b ParAnmax MSe;
lines;
Seed  1 D -4.66 .39 157.8 6.95
Seed  2 B -3.39 .35 130.3 8.08
Seed  3 C -2.96 .40 116.5 2.80
Seed  4 A -4.45 .52  94.4 2.65
Seed  5 D -1.57 .19 279.7 9.80
Seed  6 B -4.99 .40 131.8 1.46
Seed  7 A -4.43 .19 263.5 2.36
Seed  8 C -4.42 .29 179.8 2.08
Seed  9 D -14.5 .51 193.1 .
Seed 11 B -3.30 .16 227.2 0.07
Seed 12 C -5.89 .30 208.5 1.36
Tree  2 B -3.34 .07 819.0 0.23
Tree  4 A -9.3  .13 395.5 0.63
Tree  5 D -7.34 .09 802.4 8.56
Tree  6 B 11.67 .20 235.6 1.45
Tree  7 A -9.35 .21 178.8 0.52
Tree  8 C -15.5 .27 199.6 2.29
Tree  9 D -7.40 .18 457.8 5.45
Tree 10 A -8.43 .20 328.8 2.80
Tree 11 B -14.3 .20 208.9 0.63
Tree 12 C -12.1 .29 139.4 0.02
RUN;
PROC SORT DATA=Ind1;
BY AGE TRT;
RUN;
proc print data=ind1;
run;
PROC MEANS DATA = Ind1 MEAN VAR;
VAR a b ParAnmax MSe;
BY AGE trt;
RUN;
proc discrim data=ind1 pcov;
class trt;
var a b ParAnmax;
by age;
run;

Proc nlmixed data=seed method=firo;
parms aA=-4.44 bA=.355 x0A=179
      aB=-3.89 bB=.303 x0B=163
      aC=-4.42 bC=.33 x0C=168
      aD=-6.9 bD=.363 x0D=210 var=3;
      a =(aA*za)+(aB*zb)+(aC*zc)+(aD*zd)+u1;
      b =(bA*za)+(bB*zb)+(bC*zc)+(bD*zd)+u2;
      x0=(x0A*za)+(x0B*zb)+(x0C*zc)+(x0D*zd)+u3;
      if (Par<x0) then mean= a + b*Par * (1-Par/(2*x0));
                  else mean= a + b*x0/2;
  model An ~ normal(mean,var);
  random u1 u2 u3 ~ normal([0,0,0],[13.9,-.29,.021,43.69,-
8.98,4680])subject=plot;
 estimate 'Interaction on b'  (bB-bA)/2 - (bD-bC)/2;
```

```
     estimate 'Main Mean of b w Ca'   (bC+bD)/2;
     estimate 'Main Mean of b wo Ca'  (bA+bB)/2;
     estimate 'Ca Effect on b'   (bC+bD)/2 - (bA+bB)/2;

     estimate 'Main Mean of b w N'  (bB+bD)/2;
     estimate 'Main Mean of b wo N' (bA+bC)/2;
     estimate 'N Effect on b'    (bB+bD)/2 - (bA+bC)/2;

     estimate 'Interaction on x0'  (x0B-x0A)/2 - (x0D-x0C)/2;
     estimate 'Main Mean of x0 w Ca'   (x0C+x0D)/2;
     estimate 'Main Mean of x0 wo Ca'  (x0A+x0B)/2;
     estimate 'Ca Effect on x0'   (x0C+x0D)/2 - (x0A+x0B)/2;

     estimate 'Main Mean of x0 w N'  (x0B+x0D)/2;
     estimate 'Main Mean of x0 wo N' (x0A+x0C)/2;
     estimate 'N Effect on x0'    (x0B+x0D)/2 - (x0A+x0C)/2;
     estimate 'x0D vsx0A'    (x0D-x0A);
    quit;


    Proc nlmixed data=tree method=firo;
    parms aA=-9.03 bA=.355 x0A=210
          aB=-1.99 bB=.157 x0B=421
          aC=-13.8 bC=.265 x0C=410
          aD=-7.37 bD=.135 x0D=630 var=3;
          a =(aA*za)+(aB*zb)+(aC*zc)+(aD*zd)+u1;
          b =(bA*za)+(bB*zb)+(bC*zc)+(bD*zd)+u2;
          x0=(x0A*za)+(x0B*zb)+(x0C*zc)+(x0D*zd)+u3;
          if (Par<x0) then mean= a + b*Par * (1-Par/(2*x0));
                      else mean= a + b*x0/2;
      model An ~ normal(mean,var);
      random u1 u2 u3 ~ normal([0,0,0],[57.7,.04,.003,-86.77,-
12.61,53931])subject=plot;
     /*estimate 'Interaction on b'  (bB-bA)/2 - (bD-bC)/2;
     estimate 'Main Mean of b w Ca'   (bC+bD)/2;
     estimate 'Main Mean of b wo Ca'  (bA+bB)/2;
     estimate 'Ca Effect on b'   (bC+bD)/2 - (bA+bB)/2;

     estimate 'Main Mean of b w N'  (bB+bD)/2;
     estimate 'Main Mean of b wo N' (bA+bC)/2;
     estimate 'N Effect on b'    (bB+bD)/2 - (bA+bC)/2;*/

     estimate 'Interaction on x0'  (x0B-x0A)/2 - (x0D-x0C)/2;
     estimate 'Main Mean of x0 w Ca'   (x0C+x0D)/2;
     estimate 'Main Mean of x0 wo Ca'  (x0A+x0B)/2;
     estimate 'Ca Effect on x0'   (x0C+x0D)/2 - (x0A+x0B)/2;

     estimate 'Main Mean of x0 w N'  (x0B+x0D)/2;
     estimate 'Main Mean of x0 wo N' (x0A+x0C)/2;
     estimate 'N Effect on x0'    (x0B+x0D)/2 - (x0A+x0C)/2;

     estimate 'x0D vs x0A'    (x0D-x0A);
     estimate 'x0D vs x0B'    (x0D-x0B);
     estimate 'x0D vs x0C'    (x0D-x0C);
     estimate 'x0C vs x0A'    (x0C-x0A);
     estimate 'x0C vs x0B'    (x0D-x0B);
     estimate 'x0B vs x0A'    (x0B-x0A);
```

```
quit;

/*

*below is expected mean parameterization;
Proc nlin data=seed1;
parms ms=-5 mss=30 theta=10; n=17; xs=15; xss=600;
m= (n-1)*(Par-xs)/(xss-xs)+1;
F=ms+(mss-ms)*(1-theta**(m-1))/(1-theta**(n-1));
model An =F;
*by plot;
quit;
*/
```
