# Supplementary material for: Photosynthetic and Growth Response of Sugar Maple (Acer saccharum Marsh.) Mature Trees and Seedlings to Calcium, Magnesium, and Nitrogen Additions in the Catskill Mountains, NY, USA
Source: PLoS One. 2015 Aug 20;10(8):e0136148. doi: 10.1371/journal.pone.0136148 (PMC4546351; doi:10.1371/journal.pone.0136148)
Supplement: S2 Table — (PDF) [file pone.0136148.s002.pdf]

S2 Table. Basal area of mature sugar maple trees for reference, N, CaMg, and N+CaMg plots in 2003, 2007, and 2011.

Reference	cm2	cm2	cm2
	2003 BA	2007 BA	2011 BA
Plot 4	50.24	51.50385	0
Plot 4	304.6507	314	339.6224
Plot 4	183.7607	211.1336	226.865
Plot 4	359.4986	359.4986	418.8839
Plot 4	475.0506	593.6563	764.1504
Plot 4	0	91.5624	103.8163
Plot 4	62.17985	63.585	73.86065
Plot 4	482.8064	514.4576	542.9767
Plot 4	260.0234	292.4047	307.7514
Plot 4	122.6563	128.6144	158.2874
Plot 4	333.1226	362.8663	418.8839
Plot 4	471.1963	530.66	580.7744
Plot 4	52.7834	0	0
Plot 4	286.3759	352.8104	408.0744
Plot 4	208.5667	229.5419	248.7194
Plot 4	555.4346	615.44	673.9147
Plot 4	1274.911	0	0
Plot 4	304.6507	346.185	376.4939
Plot 4	669.3224	669.3224	669.3224
Plot 4	655.6399	655.6399	655.6399
Plot 4	91.5624	96.71985	109.3034
Plot 4	22.8906	23.74625	0
Plot 4	265.7696	295.4426	323.4907
Plot 4	37.37385	37.37385	37.37385
Plot 4	103.8163	103.8163	103.8163
Plot 4	352.8104	386.8794	418.8839
Plot 4	534.7499	572.265	602.3227
Plot 4	63.585	63.585	0
Plot 4	114.9319	118.7627	136.7784
Plot 4	0	0	0
Plot 4	193.4947	193.4947	0
Plot 4	60.7904	65.00585	69.3626
Plot 4	88.2026	89.87465	98.4704
Plot 4	84.9056	93.26585	93.26585
Plot 4	45.3416	47.7594	52.7834
Plot 4	33.16625	33.16625	34.1946
Plot 4	67.89465	72.3456	0
Plot 4	26.4074	26.4074	26.4074
Plot 4	24.6176	24.6176	25.50465
Plot 4	107.4587	114.9319	132.665
Plot 4	36.2984	36.2984	40.6944
Plot 4	31.15665	37.37385	48.99185
Plot 4	46.54265	46.54265	46.54265
Plot 4	0	0	0
Plot 4	203.4799	218.9287	240.4063
Plot 4	333.1226	336.3647	362.8663
Plot 4	218.9287	218.9287	221.5584
Plot 4	46.54265	46.54265	46.54265

Plot 4	113.04	134.7139	156.0659
Plot 4	0	0	0
Plot 4	81.6714	102.0186	126.6127
Plot 4	0	0	0
Plot 4	122.6563	126.6127	140.9546
Plot 4	20.41785	0	0
Plot 4	26.4074	26.4074	31.15665
Plot 4	218.9287	240.4063	240.4063
Plot 7	45.3416	46.54265	46.54265
Plot 7	33.16625	35.23865	36.2984
Plot 7	467.3576	486.7079	486.7079
Plot 7	28.26	31.15665	31.15665
Plot 7	1978.231	1978.231	1978.231
Plot 7	744.6824	788.8387	798.8239
Plot 7	323.4907	326.6856	326.6856
Plot 7	683.1463	697.1114	730.2463
Plot 7	52.7834	0	0
Plot 7	356.1467	352.8104	352.8104
Plot 7	339.6224	369.6487	397.4063
Plot 7	2122.64	2130.812	2171.907
Plot 7	1505.975	1505.975	1505.975
Plot 7	320.3114	386.8794	420.6992
Plot 7	31.15665	36.2984	38.465
Plot 7	0	0	0
Plot 7	224.2039	226.865	240.4063
Plot 7	580.7744	637.6163	690.1112
Plot 7	1748.854	1748.854	1748.854
Plot 7	23.74625	23.74625	0
Plot 7	171.9464	178.9879	184.9637
Plot 7	0	0	0
Plot 7	50.24	51.50385	50.86996
Plot 7	277.4504	286.3759	286.3759
Plot 7	221.5584	226.865	240.4063
Plot 7	40.6944	0	0
Plot 7	793.8234	834.2666	891.5167
Plot 7	25.50465	25.50465	26.4074
Plot 7	818.9827	824.0616	844.5344
Plot 7	326.6856	346.185	346.185
Plot 7	138.8587	143.0663	142.0085
Plot 7	735.0426	739.8547	751.9535
Plot 7	149.4954	149.4954	149.4954
Plot 7	628.6987	628.6987	671.6166
Plot 7	83.28065	83.28065	83.28065
Plot 7	542.9767	551.2663	551.2663
Plot 7	0	0	0
Plot 7	1540.555	1625.146	1711.999
Plot 7	542.9767	568.0339	568.0339
Plot 7	147.3367	153.86	162.7776
Plot 7	191.0376	0	0
Plot 7	0	0	0

Plot 7	498.5064	510.4463	593.6563
plot 10	0	0	0
plot 10	0	0	0
plot 10	0	0	0
plot 10	0	0	0
plot 10	0	0	0
plot 10	0	0	0
plot 10	0	0	0
plot 10	0	0	0
plot 10	0	0	0
plot 10	20.41785	23.74625	24.6176
plot 10	23.74625	23.74625	23.74625
plot 10	42.9866	42.9866	48.99185
plot 10	44.15625	50.24	50.24
plot 10	44.15625	47.7594	47.7594
plot 10	50.24	50.24	0
plot 10	54.07865	62.17985	62.17985
plot 10	56.71625	56.71625	60.7904
plot 10	76.93785	78.5	78.5
plot 10	76.93785	83.28065	83.28065
plot 10	94.985	103.8163	103.8163
plot 10	100.2367	102.0186	102.0186
plot 10	102.0186	102.0186	109.3034
plot 10	116.8394	120.7016	120.7016
plot 10	122.6563	0	0
plot 10	136.7784	140.9546	0
plot 10	160.5247	160.5247	160.5247
plot 10	181.3664	186.1706	188.5963
plot 10	186.1706	186.1706	188.5963
plot 10	193.4947	211.1336	211.1336
plot 10	200.96	216.3146	216.3146
plot 10	200.96	203.4799	203.4799
plot 10	211.1336	211.1336	0
plot 10	213.7163	213.7163	213.7163
plot 10	218.9287	218.9287	218.9287
plot 10	229.5419	0	0
plot 10	268.6663	277.4504	277.4504
plot 10	271.5786	271.5786	271.5786
plot 10	280.4099	283.385	310.8679
plot 10	292.4047	295.4426	295.4426
plot 10	298.4963	346.185	390.3727
plot 10	323.4907	329.8963	346.185
plot 10	329.8963	329.8963	329.8963
plot 10	336.3647	404.5027	404.5027
plot 10	379.94	379.94	393.8816
plot 10	404.5027	404.5027	404.5027
plot 10	459.7274	486.7079	494.5579
plot 10	486.7079	580.7744	580.7744
plot 10	518.4847	522.5274	555.4346
plot 10	568.0339	572.265	597.9816

plot 10	593.6563	602.3227	619.8439
plot 10	597.9816	637.6163	664.7459
plot 10	606.6794	611.0519	660.185
plot 10	619.8439	0	0
plot 10	642.0986	697.1114	754.385
plot 10	642.0986	0	0
plot 10	651.1104	664.7459	711.2179
plot 10	678.5226	683.1463	687.7856
plot 10	683.1463	715.9514	730.2463
plot 10	683.1463	697.1114	706.5
plot 10	683.1463	730.2463	744.6824
plot 10	769.0567	769.0567	803.84
plot 10	803.84	849.6919	891.5167
plot 10	813.9194	860.0539	860.0539
plot 10	813.9194	928.9376	928.9376
plot 10	818.9827	829.1563	0
plot 10	824.0616	829.1563	829.1563
plot 10	945.2107	1086.314	1098.027
plot 10	961.625	983.7306	1011.716
plot 10	1133.54	1187.87	1256
plot 10	1200.116	1281.246	1281.246
plot 10	1206.262	1281.246	1351.966
plot 10	1224.796	1332.49	1384.74
plot 10	1384.74	1391.342	1431.283
plot 10	1404.593	1411.242	1411.242
plot 10	1519.76	1519.76	1519.76
plot 10	1589.625	1682.797	1719.338
plot 10	1653.846	1756.273	1786.103
plot 10	1697.366	1915.683	1915.683
plot 10	1771.156	1923.446	1923.446
plot 10	1801.112	1846.516	1877.1
plot 10	1801.112	1915.683	1915.683
plot 10	2009.883	2171.907	2213.394
plot 10	2122.64	2122.64	2272.135
plot 10	2280.59	2479.375	2488.207
plot 10	2374.625	2374.625	2374.625
plot 10	2595.406	2695.659	2695.659
plot 10	2732.585	2732.585	2751.142
plot 10	2920.985	3195.295	3195.295
plot 10	0	860.0539	870.4787
plot 10	0	24.6176	26.4074

n = 189	2003 BA	2007 BA	2011 BA
SUM (cm2)	89103.54	81893.66	82406.22
Sum (m2)	8.910354	8.189366	8.240622

N	cm2	cm2	cm2
	2003 BA	2007 BA	2011 BA
Plot 2	65.00585	67.89465	72.3456
Plot 2	91.5624	98.4704	98.4704
Plot 2	138.8587	143.0663	162.7776
Plot 2	145.1936	158.2874	181.3664
Plot 2	147.3367	162.7776	183.7607
Plot 2	174.2779	216.3146	224.2039
Plot 2	181.3664	211.1336	234.9427
Plot 2	226.865	262.8887	320.3114
Plot 2	289.3824	298.4963	317.1479
Plot 2	542.9767	580.7744	589.3466
Plot 2	559.6187	606.6794	660.185
Plot 2	808.8719	978.1807	0
Plot 2	1831.319	1923.446	1962.5
Plot 2	2041.785	2163.656	2272.135
Plot 2	2041.785	2122.64	2417.996
Plot 2	2057.83	2171.907	2297.546
Plot 2	3185.287	3215.36	3215.36
Plot 6	1351.966	1404.593	1451.465
Plot 6	193.4947	193.4947	195.9674
Plot 6	518.4847	522.5274	542.9767
Plot 6	22.8906	26.4074	26.4074
Plot 6	30.1754	30.1754	30.1754
Plot 6	65.00585	65.00585	66.4424
Plot 6	346.185	349.4899	349.4899
Plot 6	268.6663	268.6663	295.4426
Plot 6	26.4074	28.26	30.1754
Plot 6	149.4954	169.6307	188.5963
Plot 6	165.0463	171.9464	176.625
Plot 6	103.8163	128.6144	128.6144
Plot 6	415.265	455.9359	482.8064
Plot 6	29.20985	31.15665	33.16625
Plot 6	245.9327	245.9327	245.9327
Plot 6	1704.675	1763.707	1884.785
Plot 6	1610.891	1625.146	1639.465
Plot 6	1127.582	1127.582	1139.514
Plot 6	54.07865	56.71625	59.41665
Plot 6	0	0	0
Plot 6	193.4947	203.4799	213.7163
Plot 6	510.4463	534.7499	589.3466
Plot 6	1661.06	1675.535	1690.074
Plot 6	1145.503	1145.503	1181.77
Plot 6	818.9827	880.9663	950.6664
Plot 6	352.8104	352.8104	356.1467
Plot 6	1763.707	1786.103	1801.112
Plot 6	2714.09	2714.09	2949.787
Plot 6	0	0	0
Plot 6	1954.658	1954.658	2009.883
Plot 6	76.93785	98.4704	98.4704

Plot 6	193.4947	213.7163	216.3146
Plot 6	426.1687	486.7079	522.5274
Plot 6	310.8679	333.1226	362.8663
Plot 6	63.585	63.585	65.00585
Plot 6	720.7007	735.0426	793.8234
Plot 6	1417.906	1505.975	1690.074
Plot 6	54.07865	58.0586	59.41665
Plot 6	304.6507	307.7514	317.1479
Plot 6	32.1536	0	0
Plot 6	0	0	0
Plot 6	0	0	0
Plot 6	1243.471	1287.596	1345.459
Plot 6	429.8346	429.8346	452.16
Plot 6	0	0	0
Plot 6	0	0	0
Plot 11	1326.03	1326.03	1326.03
Plot 11	580.7744	597.9816	633.1496
Plot 11	224.2039	224.2039	226.865
Plot 11	956.1379	0	0
Plot 11	373.0634	393.8816	397.4063
Plot 11	240.4063	240.4063	254.34
Plot 11	0	0	0
Plot 11	265.7696	257.1739	257.1739
Plot 11	576.5119	683.1463	793.8234
Plot 11	216.3146	218.9287	229.5419

n = 74	2003 BA	2007 BA	2011 BA
SUM (cm2)	44106.4	44756.47	45961.95
Sum (m2)	4.41064	4.475647	4.596195

Ca	cm2	cm2	cm2
	2003 BA	2007 BA	2011 BA
Plot 3	0	0	0
Plot 3	0	0	0
Plot 3	28.26	28.26	28.26
Plot 3	33.16625	33.16625	0
Plot 3	41.83265	0	0
Plot 3	45.3416	54.07865	59.41665
Plot 3	63.585	63.585	65.00585
Plot 3	66.4424	75.3914	80.07785
Plot 3	67.89465	70.84625	64.29346
Plot 3	70.84625	70.84625	74.62406
Plot 3	73.86065	86.54625	89.03666
Plot 3	78.5	76.93785	78.5
Plot 3	81.6714	98.4704	113.984
Plot 3	84.9056	100.2367	100.2367
Plot 3	84.9056	109.3034	109.3034

Plot 3	86.54625	0	0
Plot 3	89.87465	103.8163	113.04
Plot 3	89.87465	91.5624	101.1257
Plot 3	109.3034	122.6563	130.6319
Plot 3	109.3034	122.6563	136.7784
Plot 3	116.8394	132.665	145.1936
Plot 3	122.6563	134.7139	147.3367
Plot 3	122.6563	143.0663	161.6492
Plot 3	138.8587	145.1936	151.6699
Plot 3	147.3367	156.0659	167.3306
Plot 3	162.7776	174.2779	174.2779
Plot 3	176.625	181.3664	0
Plot 3	188.5963	211.1336	211.1336
Plot 3	191.0376	200.96	211.1336
Plot 3	198.4559	234.9427	234.9427
Plot 3	271.5786	295.4426	314
Plot 3	271.5786	314	397.4063
Plot 3	298.4963	393.8816	406.2866
Plot 3	304.6507	366.2496	386.8794
Plot 3	356.1467	397.4063	415.265
Plot 3	362.8663	459.7274	551.2663
Plot 3	510.4463	646.5967	711.2179
Plot 3	514.4576	534.7499	563.8184
Plot 3	602.3227	706.5	818.9827
Plot 3	673.9147	793.8234	839.3927
Plot 3	706.5	839.3927	934.3463
Plot 3	720.7007	886.2336	1017.36
Plot 3	818.9827	880.9663	918.1674
Plot 3	829.1563	1000.475	1074.665
Plot 3	934.3463	1057.309	0
Plot 3	1319.585	1431.283	1431.283
Plot 3	1319.585	1378.154	1451.465
Plot 3	1384.74	1582.568	1778.622
Plot 3	1589.625	0	0
Plot 3	1618.011	1632.298	0
Plot 3	2114.484	2163.656	2314.565
Plot 3	2272.135	2444.207	2640.74
Plot 3	2306.047	2263.697	2568.394
Plot 3	2435.455	2479.375	2595.406
Plot 3	2826	3007.814	3115.665
Plot 3	4474.696	4546.1	4546.1
Plot 8	0	0	0
Plot 8	0	0	0
Plot 8	23.74625	27.32585	28.26
Plot 8	24.6176	0	0
Plot 8	34.1946	35.23865	38.465
Plot 8	44.15625	0	0
Plot 8	45.3416	48.99185	48.99185
Plot 8	52.7834	55.3896	58.0586
Plot 8	58.0586	63.585	63.585

Plot 8	111.1639	120.7016	130.6319
Plot 8	116.8394	122.6563	132.665
Plot 8	120.7016	124.6266	124.6266
Plot 8	128.6144	151.6699	183.7607
Plot 8	132.665	138.8587	153.86
Plot 8	143.0663	153.86	153.86
Plot 8	167.3306	171.9464	181.3664
Plot 8	206.0154	221.5584	234.9427
Plot 8	226.865	237.6666	268.6663
Plot 8	245.9327	307.7514	346.185
Plot 8	251.5219	254.34	271.5786
Plot 8	314	333.1226	386.8794
Plot 8	326.6856	356.1467	376.4939
Plot 8	339.6224	0	0
Plot 8	339.6224	376.4939	426.1687
Plot 8	373.0634	379.94	418.8839
Plot 8	459.7274	498.5064	563.8184
Plot 8	526.5859	534.7499	555.4346
Plot 8	555.4346	572.265	660.185
Plot 8	798.8239	907.46	1006.087
Plot 8	886.2336	886.2336	886.2336
Plot 8	928.9376	1074.665	1074.665
Plot 8	934.3463	972.6464	1092.163
Plot 8	1006.087	1017.36	1017.36
Plot 8	1011.716	1092.163	1224.796
Plot 8	1547.518	1675.535	1734.065
Plot 8	2911.416	2949.787	3205.32
Plot 8	37.37385	39.57185	45.3416
Plot 8	0	0	0
Plot 8	628.6987	642.0986	683.1463
Plot 12	0	0	0
Plot 12	0	0	0
Plot 12	918.1674	967.1279	967.1279
Plot 12	1086.314	1086.314	1109.802
Plot 12	593.6563	660.185	0
Plot 12	1512.86	1711.999	0
Plot 12	38.465	40.6944	41.83265
Plot 12	23.74625	23.74625	23.74625
Plot 12	25.50465	25.50465	27.32585
Plot 12	1778.622	1884.785	1962.5
Plot 12	1365.029	1458.224	1478.595
Plot 12	0	0	0
Plot 12	76.93785	93.26585	98.4704
Plot 12	3597.883	3759.082	3759.082
Plot 12	1417.906	1424.587	1431.283
Plot 12	81.6714	88.2026	88.2026
Plot 12	28.26	0	0
Plot 12	160.5247	165.0463	167.3306
Plot 12	65.00585	65.00585	65.00585
Plot 12	283.385	0	0

Plot 12	62.17985	69.3626	70.84625
Plot 12	1293.963	1384.74	1464.998
Plot 12	0	0	0
Plot 12	602.3227	660.185	706.5
Plot 12	221.5584	234.9427	254.34
Plot 12	518.4847	538.8554	611.0519
Plot 12	408.0744	452.16	463.5347
Plot 12	89.87465	100.2367	103.8163
Plot 12	19.625	0	0
Plot 12	1243.471	1332.49	1351.966
Plot 12	471.1963	506.4506	506.4506
Plot 12	6190.07	6543.517	6629.804
Plot 12	444.6554	444.6554	444.6554
Plot 12	730.2463	730.2463	730.2463
Plot 12	1293.963	1568.501	1568.501
Plot 12	91.5624	94.985	94.985
Plot 12	1451.465	1464.998	1464.998
Plot 12	283.385	298.4963	298.4963
Plot 12	96.71985	96.71985	96.71985
Plot 12	2331.646	2577.383	2577.383
Plot 12	81.6714	84.9056	86.54625
Plot 12	44.15625	45.3416	51.50385
Plot 12	27.32585	28.26	31.15665
Plot 12	0	0	0
Plot 12	165.0463	181.3664	188.5963
Plot 12	78.5	78.5	81.6714
Plot 12	314	342.8959	376.4939
Plot 12	0	0	0
Plot 12	1074.665	1133.54	1249.728
Plot 12	2130.812	2409.291	2541.524
Plot 12	200.96	203.4799	216.3146
Plot 12	126.6127	126.6127	130.6319

n = 147	2003 BA	2007 BA	2011 BA
SUM (cm2)	85910.12	89256.45	88130.58
Sum (m2)	8.591012	8.925645	8.813058

**Ca + N**	cm2	cm2	cm2
	2003 BA	2007 BA	2011 BA
Plot 1	19.625	20.41785	20.41785
Plot 1	20.41785	23.74625	23.74625
Plot 1	46.54265	54.07865	63.585
Plot 1	63.585	78.5	88.2026
Plot 1	70.84625	75.3914	75.3914
Plot 1	83.28065	96.71985	96.71985
Plot 1	96.71985	103.8163	120.7016
Plot 1	105.6296	134.7139	167.3306
Plot 1	105.6296	107.4587	130.6319

Plot 1	171.9464	200.96	232.2344
Plot 1	245.9327	268.6663	304.6507
Plot 1	329.8963	422.5184	486.7079
Plot 1	362.8663	429.8346	522.5274
Plot 1	518.4847	606.6794	642.0986
Plot 1	1011.716	1011.716	1011.716
Plot 1	1057.309	0	0
Plot 1	1711.999	1711.999	1734.065
Plot 1	0	0	22.8906
Plot 5	989.2963	1017.36	1057.309
Plot 5	23.74625	31.15665	35.23865
Plot 5	304.6507	356.1467	390.3727
Plot 5	0	0	0
Plot 5	0	0	0
Plot 5	67.89465	75.3914	83.28065
Plot 5	1313.156	1313.156	1319.585
Plot 5	100.2367	100.2367	103.8163
Plot 5	111.1639	111.1639	122.6563
Plot 5	0	0	50.24
Plot 5	0	0	134.7139
Plot 5	134.7139	134.7139	134.7139
Plot 5	188.5963	206.0154	226.865
Plot 5	91.5624	91.5624	91.5624
Plot 5	67.89465	67.89465	76.93785
Plot 5	58.0586	66.4424	70.84625
Plot 5	0	0	70.84625
Plot 5	1931.226	1931.226	1931.226
Plot 5	63.585	63.585	63.585
Plot 5	75.3914	0	0
Plot 5	0	0	0
Plot 5	369.6487	393.8816	471.1963
Plot 5	36.2984	0	0
Plot 5	808.8719	808.8719	824.0616
Plot 5	2147.203	2155.422	2255.274
Plot 5	597.9816	597.9816	597.9816
Plot 5	1661.06	1682.797	1771.156
Plot 5	27.32585	27.32585	0
Plot 5	56.71625	56.71625	56.71625
Plot 5	486.7079	563.8184	637.6163
Plot 5	136.7784	136.7784	0
Plot 5	62.17985	69.3626	83.28065
Plot 5	397.4063	437.2136	437.2136
Plot 5	0	0	0
Plot 5	0	0	0
Plot 5	83.28065	83.28065	88.2026
Plot 5	346.185	376.4939	415.265
Plot 5	611.0519	646.5967	744.6824
Plot 5	149.4954	165.0463	195.9674
Plot 5	646.5967	678.5226	778.9163
Plot 5	195.9674	206.0154	245.9327

Plot 5	70.84625	94.985	122.6563
Plot 5	254.34	304.6507	383.4019
Plot 5	174.2779	178.9879	178.9879
Plot 5	286.3759	326.6856	386.8794
Plot 5	551.2663	637.6163	764.1504
Plot 5	226.865	226.865	254.34
Plot 5	38.465	38.465	38.465
Plot 5	593.6563	664.7459	778.9163
Plot 5	226.865	260.0234	314
Plot 5	2314.565	2383.268	2568.394
Plot 5	162.7776	191.0376	213.7163
Plot 5	1384.74	1384.74	1458.224
Plot 5	849.6919	886.2336	939.7706
Plot 5	870.4787	902.1299	1017.36
Plot 5	961.625	994.8776	1068.864
Plot 5	1045.816	1098.027	1193.985
Plot 5	739.8547	793.8234	891.5167
Plot 9	452.16	459.7274	459.7274
Plot 9	490.625	490.625	490.625
Plot 9	803.84	854.865	891.5167
Plot 9	530.66	580.7744	580.7744
Plot 9	637.6163	683.1463	697.1114
Plot 9	28.26	30.1754	32.1536
Plot 9	23.74625	23.74625	23.74625
Plot 9	415.265	433.5163	433.5163
Plot 9	1451.465	1618.011	1625.146
Plot 9	1256	1378.154	1451.465
Plot 9	298.4963	0	0
Plot 9	530.66	530.66	568.0339
Plot 9	803.84	803.84	824.0616
Plot 9	2205.065	2230.099	2461.76
Plot 9	38.465	38.465	45.3416
Plot 9	23.74625	23.74625	23.74625
Plot 9	96.71985	109.3034	114.9319
Plot 9	186.1706	186.1706	188.5963
Plot 9	314	346.185	346.185
Plot 9	744.6824	759.2599	778.9163
Plot 9	29.20985	29.20985	29.20985
Plot 9	24.6176	24.6176	25.50465
Plot 9	120.7016	120.7016	126.6127
Plot 9	983.7306	1098.027	1115.713
Plot 9	329.8963	329.8963	329.8963
Plot 9	386.8794	390.3727	400.9466
Plot 9	44.15625	48.99185	50.24
Plot 9	854.865	928.9376	956.1379
Plot 9	38.465	51.50385	51.50385
Plot 9	206.0154	218.9287	218.9287
Plot 9	46.54265	47.7594	47.7594
Plot 9	764.1504	813.9194	854.865
Plot 9	132.665	132.665	132.665

Plot 9	167.3306	167.3306	167.3306
Plot 9	939.7706	1011.716	1011.716
Plot 9	880.9663	928.9376	967.1279
Plot 9	669.3224	683.1463	683.1463
Plot 9	2171.907	2098.219	2130.812
Plot 9	1109.802	1163.566	1218.603
Plot 9	206.0154	211.1336	216.3146
Plot 9	798.8239	803.84	818.9827
Plot 9	94.985	96.71985	100.2367
Plot 9	19.625	22.05065	26.4074
Plot 9	33.16625	34.1946	35.23865
Plot 9	2348.791	2444.207	2444.207
Plot 9	48.99185	50.24	56.71625
Plot 9	880.9663	896.8154	896.8154
Plot 9	245.9327	245.9327	262.8887
Plot 9	356.1467	356.1467	356.1467
Plot 9	994.8776	1063.078	1127.582
Plot 9	138.8587	149.4954	162.7776
Plot 9	30.1754	30.1754	30.1754
Plot 9	113.04	114.9319	114.9319
Plot 9	1711.999	1711.999	1711.999
Plot 9	26.4074	32.1536	40.6944
Plot 9	2658.983	2658.983	2714.09
Plot 9	0	0	0
Plot 9	2025.802	2098.219	2155.422
Plot 9	1519.76	1596.698	1823.743
Plot 9	66.4424	0	0
Plot 9	21.2264	26.4074	31.15665
Plot 9	1861.777	2001.946	2033.786
Plot 9	983.7306	1074.665	1080.482
Plot 9	29.20985	30.1754	35.23865
Plot 9	44.15625	44.15625	44.15625
Plot 9	102.0186	103.8163	109.3034
Plot 9	0	0	0
Plot 9	655.6399	701.7979	711.2179
Plot 9	19.625	21.2264	0
Plot 9	813.9194	813.9194	813.9194
Plot 9	41.83265	44.15625	47.7594
Plot 9	1319.585	1319.585	1391.342
Plot 9	673.9147	706.5	754.385
Plot 9	1458.224	1533.607	1618.011
Plot 9	928.9376	989.2963	1028.695
Plot 9	213.7163	221.5584	0
Plot 9	52.7834	52.7834	52.7834
Plot 9	52.7834	46.54265	54.07865
Plot 9	673.9147	683.1463	683.1463
Plot 9	188.5963	188.5963	188.5963
Plot 9	2409.291	2514.795	2631.642
Plot 9	390.3727	415.265	429.8346
Plot 9	107.4587	107.4587	111.1639

Plot 9	602.3227	619.8439	651.1104
Plot 9	317.1479	323.4907	329.8963
Plot 9	268.6663	274.5067	314
Plot 9	907.46	956.1379	1057.309
Plot 9	122.6563	124.6266	128.6144
Plot 9	989.2963	1034.387	1092.163
Plot 9	891.5167	983.7306	1017.36
Plot 9	669.3224	692.4407	720.7007
Plot 9	36.2984	38.465	41.83265
Plot 9	29.20985	30.1754	31.15665
Plot 9	1771.156	1970.358	1970.358
Plot 9	286.3759	292.4047	289.3824
Plot 9	35.23865	35.23865	38.465
Plot 9	1115.713	1133.54	1200.116
Plot 9	1045.816	1080.482	1080.482
Plot 9	0	0	0
Plot 9	208.5667	224.2039	229.5419
Plot 9	1568.501	1668.29	1801.112
Plot 9	94.985	102.0186	109.3034
Plot 9	52.7834	52.7834	62.17985
Plot 9	1268.591	1319.585	1397.959
Plot 9	32.1536	32.1536	32.1536
Plot 9	48.99185	52.7834	62.17985
Plot 9	31.15665	31.15665	34.1946
Plot 9	715.9514	764.1504	778.9163
Plot 9	138.8587	140.9546	143.0663
Plot 9	120.7016	120.7016	120.7016
Plot 9	972.6464	1074.665	0
Plot 9	803.84	839.3927	912.8059
Plot 9	352.8104	352.8104	764.1504
Plot 9	22.8906	25.50465	30.1754
Plot 9	739.8547	844.5344	844.5344
Plot 9	808.8719	829.1563	0
Plot 9	58.0586	63.585	70.84625
Plot 9	211.1336	216.3146	221.5584
Plot 9	23.74625	25.50465	30.1754
Plot 9	1243.471	1338.967	1404.593
Plot 9	314	314	307.7514
Plot 9	769.0567	769.0567	0
Plot 9	615.44	646.5967	664.7459
Plot 9	22.05065	22.8906	23.74625
Plot 9	1589.625	1625.146	1778.622
Plot 9	94.985	94.985	0
Plot 9	1675.535	1719.338	359.4986
Plot 9	373.0634	373.0634	437.2136
Plot 9	362.8663	369.6487	369.6487
Plot 9	433.5163	433.5163	437.2136
Plot 9	286.3759	0	0
Plot 9	23.74625	23.74625	27.32585
Plot 9	0	26.4074	30.1754

Plot 9	0	19.625	27.32585
Plot 9	0	23.74625	23.74625
Plot 9	0	0	20.41785
Plot 9	0	0	21.2264
Plot 9	0	0	21.2264
Plot 9	0	0	22.8906
Plot 9	0	0	22.05065
Plot 9	0	0	19.625

n=217	2003 BA	2007 BA	2011 BA
SUM (cm2)	104878.9	107664.7	109014.2
Sum (m2)	10.48789	10.76647	10.90142
